# Determinants of dietary practice among pregnant women at the public hospitals in Bench-Sheko and Kaffa Zones, Southwest Ethiopia

**DOI:** 10.1186/s40795-022-00588-7

**Published:** 2022-08-24

**Authors:** Abel Girma, Amare Genetu, Ermias Ayalew, Dawit Getachew

**Affiliations:** 1 School of Public Health, College of Medicine and Health Science, Mizan-Tepi University, Mizan-Aman, Ethiopia; 2 Department of Midwifery, College of Medicine and Health Science, Mizan-Tepi University, Mizan-Aman, Ethiopia; 3 Departements of Nursing, College of Medicine and Health Science, Mizan-Tepi University, Mizan-Aman, Ethiopia

**Keywords:** Antenatal care, Bench-Sheko zone, Dietary practice, Kaffa zone, Pregnant women

## Abstract

**Backgrounds:**

The frequency of poor dietary practice due to inappropriate dietary habits is higher during pregnancy compared to any other stage of the life cycle. Suboptimal dietary practices during pregnancy can increase the risk of intrauterine growth restriction, low birth weight, anemia, prenatal and infant mortality, and morbidity. Therefore, this study aimed to determine the dietary practice and associated factors among pregnant women at the public hospitals of Bench-Sheko and Kaffa zone.

**Methodology:**

An institutional-based cross-sectional study design was conducted among 566 pregnant women who attended antenatal care at the public hospitals of the Bench-Sheko and Kaffa zones. A systematic random sampling technique was employed to select the study units. The data were entered into Epi Data 3.1 and exported to Statistical Package for Social Science (SPSS) version 21 software for further analysis. Both Binary and Multivariable logistic regression analyses were used to examine the association between dependent and independent variables. The Crude Odd Ratio (COR) and Adjusted Odd Ratio (AOR) with 95% Confidence interval (CI) were calculated and the variable with *P*-value < 0.05 was considered statistically significant.

**Result:**

According to this study, only 23.7% (95% CI: 20.1, 27.4) of the study participants had a good dietary practice. The urban residents (AOR = 2.64; 95% CI:1.18, 5.92), monthly income of > 2000ETB (AOR = 2.47; 95% CI: 1.31,4.65), having nutrition information (AOR = 2.5; 95% CI: 1.14,5.52), good dietary knowledge (AOR = 2.79; 95% CI: 1.48,5.27), mothers occupation of employer (AOR = 1.88; 95% CI: 1.04,3.42) and a family size < 5 (AOR = 3.37; 95% CI: 1.32,8.65) were determinate of dietary practice.

**Conclusion:**

Generally, the prevalence of good dietary practice is suboptimal in the study area. Urban residency, monthly income > 2000ETB, good dietary knowledge, having nutrition information, family size < 5, and government employed mothers were the predictors of the good dietary practice in the Bench-Sheko and Kaffa zone. Therefore, providing in-service training for health professionals and assigning nutritionist to each public hospital should be done to provide health and nutrition education; and strengthen the existed nutrition counseling service for pregnant women. Moreover, the government should create sustainable income-generating activities for pregnant women.

## Introduction

Pregnant women who eat the right amount of macronutrients and micronutrients can have better pregnancy outcomes and better health for themselves and their babies [1]. The promotion of women health and prevention of health care from womb to throughout the life process critically determine women health status and the cycle of malnutrition [2]. During pregnancy, the recommended nutrient intake for most nutrients increased [3], However, most pregnant women fail to meet the World Health Organization’s (WHO) recommended nutrient intake levels [4, 5] Moreover, compared to any other time in the life cycle, the frequency of poor dietary practice due to an inappropriate dietary habit is higher during pregnancy [6]. In middle and low-income countries,the most common diet feature during pregnancy was imbalanced macronutrients, insufficient micronutrient intakes, and a predominance of plant-based food intake [7].

Multiple-micronutrient deficiencies are common in low- and middle-income countries, and they can worsen during pregnancy, potentially leading to adverse outcomes for pregnant women [7]. Poor dietary practice during pregnancy increases the risk of intrauterine growth restriction, low birth weight, preterm birth, anemia, increased infection, congenital disability, pre-eclampsia, prenatal and infant mortality, and morbidity [8].

Complex biological, physiological, psychological, economic, social, cultural beliefs, food taboos, and environmental factors contribute to poor dietary practices and undernutrition in women [9–11]. Women's nutritional status in middle and low-income countries is influenced by factors such as food insecurity, a lack of access to health care, a heavy workload, and poor sanitation and hygiene [12]. Another thing that has been found in different studies is that socioeconomic and demographic factors, as well as obstetric and pregnancy-related factors; and dietary related factors like knowledge, attitude were the main factors that had a big impact on pregnant women's dietary habits [6, 13–18].

Pregnant women should follow a variety of context-based nutritional intervention strategies to improve their nutritional status during pregnancy, according to various organizations and scholars [19–22]. Undernutrition has an intergenerational effect, and Ethiopia's government has developed a 2016 intervention strategy called the National Nutrition Program II (NNPII) that targets a thousand critical days and launched food and nutrition policy in 2018 to address food and nutrition insecurity that considered one thousand critical day as one of its implementation approach [23, 24]. However, macronutrient and micronutrient deficiencies among pregnant women are the most common public health issues in Ethiopia. Ethiopian women consume a total of 13%, 50%, and 82% of the recommended amount of iron, zink, and vitamin-A, respectively, while women in the South Nation, Nationality, and Peoples Region (SNNPR) consume 32.6%, 75.1%, and 41.3% of these nutrients [25]. Moreover, the number of pregnant women in Ethiopia who eat a healthy diet ranges from 19.9 to 78% [14, 26]. In addition, previous Ethiopian studies have primarily focused on the nutritional status of pregnant women, and there is inconsistent evidence about the dietary practices of Ethiopian pregnant women [15]. Additionally, there is no prior research in the study area. As a result, the purpose of this study was to determine the prevalence of dietary practices and their associated factors among pregnant women receiving antenatal care (ANC) at public hospitals in the Bench-Sheko and Kaffa zones of Southwest Ethiopia.

## Methods and materials

### Study area

Bench- Sheko zone is found in Southwest Region which is the new formed region of Ethiopia. Its administrative center is Mizan-Aman Town which is located 562 km far from the capital city of Addis Ababa, Ethiopia. The total population of the zone for the year 2017 is estimated to be 613,146, of whom 303,321 were male and 309,825 were females [27]. The zone has 6 districts and one town administration. It has 26 public health centers and 1 teaching hospital. The hospital had 540 antenatal care (ANC) attendants which estimated based on the 3- month average ANC follow-up women before data collection started in 2021. Kaffa zone is one of the 5 zones in Southwest Region which is located in southwest Ethiopia. Administratively, the zone has 10 districts and one town administration. Its administrative center is Bonga town which is far 468 km from the capital city Addis Ababa. The total population of the zone for the year 2017 is estimated to be 1,102,278, of whom 541,682(49.14%) are male and 560,596(50.86%) were female [27]. The kaffa zone has one general hospital, one primary hospital, and 43 public health centers. Based on a 3-month estimated average ANC follow –up of women before a data collection, there have been 490 and 401 ANC attendants in the general hospital and primary hospital respectively, by 2021.

### Study design and period

An institutional-based cross-sectional study design was conducted from May 20-June 30 in 2021.

### Source population

All pregnant women who attended ANC at the public hospitals in Bench-Sheko and Keffa zones.

### Study population

Systematically selected study participants from those who came to the public hospitals for ANC follow-up during a study period.

### Study unit

Is the pregnant women from whom information was collected.

### Inclusion and exclusion criteria

#### Inclusion criteria

The study subjects were all pregnant mothers who were attending public hospitals for ANC and who lived for at least 6 months in the study area.

#### Exclusion criteria

Mothers too sick or mentally not stable to respond to questions.

### Sample size and sampling technique

#### Sample size determination

In this study, the single population proportion formula was used to figure out the sample size. The prevalence of good dietary practices is thought to be 33.9% [28], 5% margin of error, 95% confidence level, design effect of 1.5, and a none response rate of 10%. Based on this, the actual sample size was: *n* = (zα/2)2 p (1-p)/d2 = (1.96)^2^*0.339(1–0.339)/0.05^2^, *n* = 344. Then, the design effect of 1.5 was considered (344*1.5) and became 516. A 10% none response rate was considered, then the minimum sample size required became: 516 + (516*10%) = 568. Hence, the final sample size of this study became 568.

#### Sampling technique and procedure

First, the total sample sizes were proportionally allocated to each public hospital of the Bench-Sheko and Kaffa zone (Mizan-Tepi University Teaching hospital, Gebretsadek-Shawo General hospital and Wacha primary hospital), that were all hospitals included in the study. The total population size of each public hospital was estimated by using the average number of clients attending ANC for the last 3-months based on their registration card, before the data collection period. Then, the sampling interval (K^th^) was calculated by using the formula of K = N/n. Next, prospectively every K^th^ (roughly 2) person was selected by using a systematic random sampling technique until the desired sample size was attained from each hospital.

### Study variables

The dependent variable in this study was maternal dietary practices during pregnancy, whereas the independent variables were age, marital status, religion, family size, occupation, education, income level, radio, trimester, number of pregnancy, number of live birth, pregnancy interval, number of ANC visit, food security status, residency, dietary information, dietary knowledge, dietary attitude, and history of illness.

### Data collection procedures

Structured and semi-structured questionnaires were administered by trained health professionals (Nurses and Midwifes) to collect the data. Data on socioeconomic status, pregnancy-related factors, and household food insecurity status were collected. The pregnant women's knowledge and attitudes about food were also assessed.

Pregnant women's dietary practices were assessed using a questionnaire adapted from previous literature [6, 17] and FAO Guideline [29]. Pregnant women's dietary practices were assessed retrospectively using a measurement of method of short food intake checklist that asked whether a particular list of food was consumed the previous 24 h with the answer being Yes or No; nutrition-behaviors checklists measurements, which are used to assess specific observable behaviors or practices that are some important practices for nutrition during pregnancy but cannot be assessed through food intake measurements [29] and meal frequency. Ten items were used to assess pregnant women's dietary practices. The dietary practices score was calculated by adding the responses to each question. Each question received one point if the response was correct, favorable, or healthy for dietary practices, and zero points if the response was incorrect, unfavorable, or unhealthy for dietary practices during pregnancy [6, 15, 28, 30]. Finally, participants were classified as having poor dietary practices and as having good dietary practices [6, 31, 32].

Ten open-ended questions adapted from a previous study were used to assess dietary knowledge [16, 17], which sought to assess pregnant women's knowledge of nutrition-related topics and recommended dietary advice during pregnancy [33]. Its reliability was evaluated in this study and revealed a Cron-bach Alpha of 0.92. The items assessing nutritional knowledge were scored on a dichotomous scale, with 0 indicating ignorance and 1 indicating knowledge. A correct response was coded as 1 and an incorrect response as 0. Then, the total score was obtained by summation of each score. Finally, nutritional knowledge level was categorized as knowledgeable and not knowledgeable [16, 28].

Pregnant women's attitudes toward their dietary practices during pregnancy were assessed using questioners adapted from previous studies and conceptualized for the local context [16, 17]. Maternal dietary attitudes were elicited through the use of nine questions in this study. Respondents were asked to rate their favorableness or unfavorability toward a particular dietary regimen during pregnancy. The reliability of the attitude questions was checked and showed a Cronbach Alpha of 0.84. The pregnant women were given one mark if the answers were favorable attitude for dietary practices and while zero scores were given if the responses were unfavorable [16, 31]. After the summation of the score, the respondent was categorized as favorable attitude and unfavorable attitude [16]

The Household Food Insecurity Access Scale (HFIAS) was used to measure the level of food insecurity in each household. This is a structured, standardized, and validated tool that was mostly made by FANTA [34, 35] and a scale is a valid tool in measuring household food insecurity among both rural and urban areas of Ethiopia [36]. Food insecurity (access) is measured using nine questions that represent increasing levels of severity and nine "frequency-of occurrence" questions that ask about the changes in diet or food consumption patterns that households have made due to limited resources in the previous 30 days. Participants received a score ranging from 0 to 27 based on their answers to nine questions and the frequency with which they occurred over the previous 30 days. A lower HFIAS score indicates better access to food and less household food insecurity, whereas a high HFIAS score indicates a lack of access to food and a lack of food insecurity [34].

### Data quality assurance

In order to ensure the validity of the data, a pre-test was conducted among 5% of the study participants. Amharic and English translations of the final questionnaire were done in order to better understand the respondents' native language. All data collectors received two days of in-depth training on the instruments and methods for data collection, as well as the ethical issues involved in conducting a research project. Throughout the data collection period, supervisors checked the collected data for completeness, accuracy, and consistency, and the principal investigator was in charge of overall supervision. A comparison of two data cells was made using double data entry.

### Data analysis methods

After verifying that all data were complete and consistent internally, they were coded and entered into the Epi Data 3.1 version computer software package, which was then exported to the Statistical package for social science (SPSS) version 21 software for further analysis. Percentage, frequency, mean, and standard deviation were calculated for the descriptive statistical analyses. We used bivariable logistic regression to examine the relationship between the dependent and independent variables. The variables with a *P*-value < 0.25 during bivariable logistic regression analysis were considered for multivariable logistic regression models to control all possible confounders and to identify factors independently associated with the dietary practice of pregnant women. The Crude Odds Ratio (COR) and Adjusted Odds Ratio (AOR) with 95 percent confidence intervals (CI) were calculated to determine the strength and direction of association between dependent and independent variables. Finally, the variable with (*p*-value < 0.05) in multivariable logistic regression analysis was considered statistically significant. Multicollinearity between independent variables was checked by using standard error (SE) and variables of SE > 2 were dropped from the analysis. The model fitness was tested by Hosmer- Lemeshow for the goodness of fit and model fitted was considered at Hosmer–Lemeshow *P*-value > 0.05.

### Operational definition

#### Dietary practice

This is the observable action of the mother that could affect her nutrition such as eating, feeding, cooking, and selecting foods. The study participants were classified as having poor dietary practices if they correctly answered < 75% of dietary practice questions and good dietary practices were if they correctly answer ≥ 75% of questions [31, 32].

#### Knowledge

Is awareness and understanding that one has gained on nutrition during pregnancy through learning and practice. The pregnant women were considered to be knowledgeable if they were correctly answered ≥ 70% of the total knowledge assessing questions and non-knowledgeable if respondents score < 70% on the knowledge questions [16, 33].

#### Attitudes

Pregnant women’s feeding or eating behavior is influenced by their emotions, motivations, perceptions, and thoughts. In this study, the favorable attitude was if the respondent's attitude score > the median and while unfavorable if the respondents ‘attitude scores were ≤ the median [16].

#### Food secure

Households those experiences none of the food insecurity (access) conditions or just experience worry, but rarely in the past 4 weeks were labeled as ‘food secured or food secure households who were experienced fewer than the first 2 food insecurity indicators.

#### Food insecure

The inability of households to access sufficient food at all times to lead an active and healthy life (includes all stages of food insecurity; mild, moderate, and severe) [34]. A household that was an experience from 2–10, 11–17, and > 17 food insecurity indicators were considered as mildly, moderately, and severely food insecure households, respectively.

## Results

### Socio-demographic and economic characteristics of pregnant women

A total of 566 study participants have participated in this study, which made a response rate of 99.6%. The mean (± SD) age of the study participants was 27.01(± 4.86), and about 357 (63.1%) were between the age group of 25 and 34 years. Around 267(47.2%) pregnant women were protestant religion followers and 228(40.3%) were Kaffa by ethnicity. Almost all, 552(97.5%) pregnant women are married, and 371 (65.5%) of pregnant women were urban dwellers. Among the study participants, 247 (43.6%) of pregnant women had less than 1000 birr monthly income, and 382 (67.5%) had TV/ Radio in their home. In this study, 104(18.4%) respondents were from food-insecure households (Table 1).

**Table 1:** Socio-Demographics characteristics of the study participants at the public hospitals of Bench-Sheko and Kaffa zone, southwest Ethiopia 2021, (*N* = 566)

Variables		Frequency (N)	Percent (%)
Age	16–24	167	29.5
	25–34	357	63.1
	≥ 35	42	7.4
Religion	Orthodox	224	39.6
	Protestant	267	47.2
	Muslim	73	12.9
	Others	2	0.4
Ethnicity	Bench	148	26.1
	Kaffa	228	40.3
	Sheka	26	4.6
	Amhara	132	23.3
	Others	32	5.7
Marriage	Married	552	97.5
	Others	14	2.5
Residency	Urban	371	65.5
	Rural	195	34.5
Mother education	No formal education	173	30.6
	Primary	170	30.0
	Secondary	93	16.4
	College and above	130	23
Husband education	No formal education	125	22.1
	Primary	142	25.1
	Secondary	111	19.6
	College and above	188	33.2
Mother occupation	Housewife	327	57.8
	Merchant	79	14.0
	Employers	124	21.9
	Others	36	6.4
Husband occupation	Farmer	164	29.0
	Merchant	173	30.6
	Employer	160	28.3
	Others^a^	69	12.2
Family size	< 5	437	77.2
	≥ 5	129	22.8
Family monthly income	< 1000EBR	247	43.6
	1000-2000ETB	71	12.5
	> 2000ETB	248	43.8
TV/ Radio	Yes	382	67.5
	No	184	32.5
Mobile	Yes	404	71.4
	No	162	28.6
Households food security	Secure	462	81.6
	Insecure	104	18.4

^a^Daily labor; *TV* Television, ETB Ethiopian Birr

### Obstetric and pregnancy-related characteristics of the study participants

It was found that 471 (83.2%) of study participants had less than or equal to two live birth, and only 65(11.5%) had four and above ANC follow-up for the current pregnancy. Among the studied pregnant women, 269 (47.5%) were in their second trimester of pregnancy. Around 335(59.25) pregnant women had less than or equal to two pregnancies in their lifetime. Around 414(73.1) had nutrition information, and only 103(18.1%) had a history of illness (Table 2).

**Table 2:** Obstetric and pregnancy-related characteristics of study participants at the public hospitals of Bench-Sheko and Kaffa zone, southwest Ethiopia 2021 (*N* = 566)

Variables		Frequency (N)	Per cent (%)
Trimester	First trimester	58	10.2
	Second trimester	269	47.5
	Third trimester	239	42.2
Total number of pregnancy	≤ 2	335	59.2
	3–4	163	28.8
	≥ 5	68	12.0
Total number of live birth	≤ 2	471	83.2
	3–4	82	14.5
	≥ 5	13	2.3
Pregnancy interval	≤ 2	352	62.2
	3–5	138	24.4
	> 5	76	13.4
Number of ANC visit	One	188	33.2
	Two	210	37.1
	Three	103	18.2
	Four and above	65	11.5
History of illness	Yes	103	18.2
	No	463	81.8
Nutritional information	Yes	414	73.1
	No	152	26.9
Source of nutrition information	Health professionals	348	61.5
	Family	23	4.1
	Media	32	5.7
	Friends	7	1.2
	Others	4	0.7

### Dietary knowledge of the study participants

According to this study, around 294 (51.9%) of the study participants had good dietary knowledge. The mean score of dietary knowledge was 5.87(± 3.78 SD). Around 317(56%) and 226(39.9%) of the respondent didn’t know balanced diets and whether the pregnant diet differs from a then-pregnant diet. About 252(44.5%), 198(35%), and 240(42.4%) of the study participants didn’t know protein, iron, and vitamin source foods respectively and while around 429 (75.8%) and 375 (66.3%) knew about the danger of malnutrition on mothers and fetus respectively.

### The dietary attitude of study participants

**Fig. 1:**
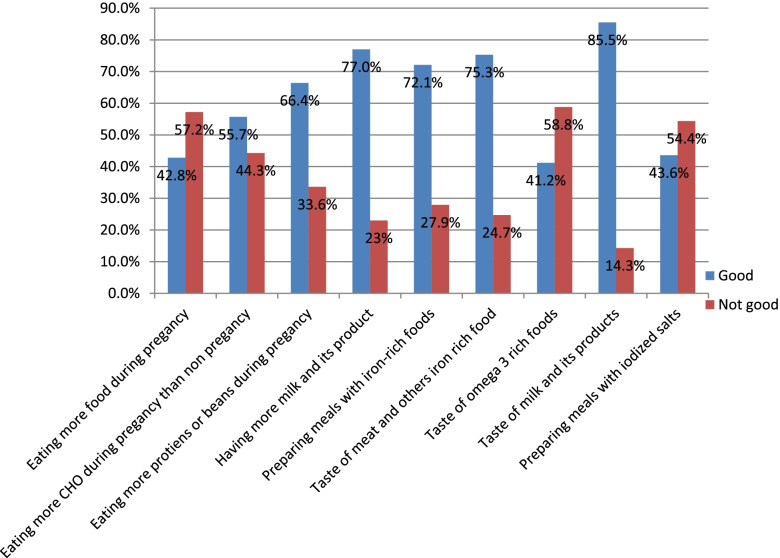
Dietary attitude of the study participants in Bench-Sheko and Kaffa zone, southwest Ethiopia 2021 (*N* = 566)

In this study, around 308 (45.6%) of the respondent had a favorable attitude toward dietary practice during pregnancy. The mean score of dietary attitude score was 5.85(± 2.7 SD). More than half, (57.2%) of the respondents considered as eating more food during pregnancy is not good. About, 251(44.3%) considered eating more carbohydrate food during pregnancy is not good (Fig. 1).

### The dietary practice of study participants

Out of 566 respondents, only 23.7% (95% CI: 20.1, 27.4) had good dietary practice. The mean score of dietary practice was 5.42(± 1.99 SD). Regarding the specific dietary practice, about 303(53.5%) and 261 (46.1%) of the respondents had the habit of eating a snack between meals and eating an additional meal during pregnancy. Around 8(1.4%) and 50(8.8%) of the respondent's meal frequency were one and two times a day respectively and while 247(43.6%) were had three times a day. About one-fourth,142 (25.1%) of the respondents had the habit of avoiding certain foods items during pregnancy, out of this, about (48.6%) and (32.4%) of were due to religious and personal dislike reasons respectively and while none of the respondents reported due to cultural reason. Around 220(38.9%) of the respondent had the habit of eating animal’s source foods daily (Table 3).

**Table 3:** Dietary practice of the study participants at the public hospitals of Bench-Sheko and Kaffa zone, southwest Ethiopia 2021 (*N* = 566)

Variables	Dietary practice	Frequency (N)	Percent (%)
Following specific dietary regimen during pregnancy	Yes	62	11
	No	504	89
Eating more carbohydrate source foods daily	Yes	203	35.9
	No	363	64.1
Eating animal source foods daily like meat, milk, and others	Yes	220	38.9
	No	346	61.1
Eating fresh fruits daily like orange, mango	Yes	389	68.7
	No	177	31.3
Eating fresh vegetables daily	Yes	423	74.7
	No	143	25.3
Eating snacks between main meals daily	Yes	303	53.5
	No	263	46.5
Taking iron-folic supplements daily	Yes	466	82.3
	No	100	17.7
Taking additional meals per day during pregnancy	Yes	261	46.1
	No	305	53.9
Number of meal frequency	One	8	1.4
	Two	50	8.8
	Three	247	43.6
	Four and above	261	46.1
Monitoring weight during pregnancy	Yes	426	75.3
	No	140	24.7
Skipping meals during pregnancy	No	443	78.3
	Yes	123	21.7
Types of skipped meals	Dinner	57	46.3
	Breakfast	30	24.4
	Lunch	36	29.3
Avoiding any foods or diet during pregnancy	No	424	74.9
	Yes	142	25.1
Reason for avoiding foods/diet during pregnancy	Religion	69	48.6
	Culture	0	0
	Avoid big baby	14	9.8
	Labor difficulty	13	9.2
	Dislike	46	32.4
Overall dietary practice status	Good (≥ 75%)	134	23.7
	Poor (< 75%)	432	76.3

### Factors associated with the dietary practice of pregnant women

In multiple logistic regression analysis the variables of residency, monthly income, dietary knowledge, nutrition information, mother occupation, and family size have shown a significant association with the outcome variable (*P* < 0.05). The urban residents were 2.64 times more likely to have good dietary practice than the rural residents (AOR = 2.64; 95% CI: 1.18, 5.92). The study participants with a monthly income of greater than 2000 ETB were 2.47 times more likely to have good dietary practice than those who earn with an average monthly income of less than 1000 ETB (AOR = 2.47; 95% CI: 1.31,4.65). The respondents who have nutrition information and good dietary knowledge were 2.5 and 2.79 times higher chance to have good dietary practice than their counterparts (AOR = 2.5; 95% CI: 1.14,5.52) and (AOR = 2.79; 95% CI: 1.48,5.27) respectively. The mothers of government employers were 1.88 times more likely to have good dietary practice than the household occupation respondents(AOR = 1.88; 95% CI: 1.04,3.42) and also the respondent with a family size of less than 5 were 3.37 times more likely to have good dietary practice than greater than 5 family size (AOR = 3.37; 95% CI: 1.32,8.65) (Table 4).

**Table 4:** Binary and Multiple logistic regression analysis for factors associated with dietary practice among pregnant women at public hospitals of Bench-Sheko and Kaffa zone, Southwest Ethiopia, 2021 (*N* = 566)

Variables	Categories	Dietary practice		COR(95%CI)	AOR(95%CI)
		Good	Poor		
Residence	Urban	125(33.7%)	246(66.3%)	10.50(5.20,21.21)**	2.64(1.18,5.92)***
	Rural	9(4.6%)	186(95.4%)	1	1
Family size	≤ 4	126(28.8%)	311(71.2%)	6.13(2.91,12.91)**	3.37(1.32,8.65)**
	≥ 5	8(6.2%)	121(93.8%)	1	1
Monthly income	< 1000	19(7.7%)	228(92.3%)	1	1
	1000–2000	14(19.7%)	57(80.3%)	2.95(1.39,6.23)**	2.24(0.95,5.28)
	> 2000	101(40.7%)	147(59.3%)	8.25(4.84,14.04)**	2.47(1.31,4.65)***
Dietary knowledge	Poor	18(6.6%)	254(93.4%)	1	1
	Good	116(39.5%)	178(60.5%)	9.20(5.40,15.66)**	2.79(1.48,5.27)***
Nutrition information	Yes	124(30%)	290(70%)	6.07(3.09,11.92)**	2.51(1.14,5.52)*
	No	10(6.6%)	142(93.4%)	1	1
Dietary attitude	Unfavorable	38(12.3%)	270(87.7%)	1	1
	Favorable	96(37.2%)	162(62.8%)	4.21(2.76,6.43)**	1.65(0.99,2.74)
Mother occupation	Housewife	37(11.3%)	290(88.7%)	1	1
	Merchant	39(33.9%)	76(66.1%)	4.02(2.40,6.74)**	1.63(0.88,3.04)
	Employer	58(46.8%)	66(53.2%)	6.89(4.21,11.26)**	1.88(1.04,3.42)*
Total number of pregnancy	≤ 2	88(26.3%)	247(73.7%)	11.76(2.82,49.00)**	1.46(0.28,7.72)
	3–4	44(27%)	119(73%)	12.20(2.87,51.95)**	1.91(0.37,9.82)
	≥ 5	2(2.9%)	66(97.1%)	1	1

^***^ = *P* < 0.001; ** = *P* < 0.01; * = *P* < 0.05

*Abbreviation*: *COR* Crude odds ratio, *AOR* adjusted odds ratio

## Discussions

Good dietary practice during pregnancy is one of the most determinant factors for the long-term health and nutritional status of mothers and their fetuses. Therefore, this study aimed to assess dietary practice and associated factors among pregnant women at public hospitals of Bench-Sheko and Kaffa zone, Southwest Ethiopia. According to this study's findings, only 23.7% (95% CI: 20.1, 27.4) of the study participants had good dietary practices. This finding is nearly similar to the study finding from Ambo, 26.9% [13]. However, it is higher than the study findings from the West Gojjam Zone, Northwest Ethiopia which showed only 19.9% [14] of pregnant women had good dietary practice. The possible explanation for this prevalence discrepancy might be due to measurement tools variation, the study in West Gojjam zone were use dietary diversity score, food variety score, animal source food and meal frequency and while this study used a short food intake checklist, nutrition-behaviors checklist and meal frequency to determine dietary practice. Moreover; this could be due to study setting difference, where the study in the West Gojjam zone was conducted among rural residents only while our study is conducted in both urban and rural settings which could have good nutrition information and counseling service. In contrary, it is lower than with the study finding from the Gedeo zone of southern Ethiopia, Guto Gida woreda of East Wollega, and Misha woreda which revealed that 32.2% [17], 33.9% [28] and 29.5% [18] of pregnant women had good dietary practice during pregnancy respectively. Furthermore, it is much lower than the study findings which conducted in Mettu Karl Hospital of southwest Ethiopia, Bahir-Dar, Gondar town of Northwest Ethiopia, Adis Ababa, and Horo Guduru Wolega zone which showed 78% [26], 39.3% [6], 40.1% [15], 34.5% [16] and 74.6% [37] of the pregnant women had good dietary practice respectively. The possible explanation for this discrepancy might be due to socio-demographic and economic factors, seasonal variation of food production and consumption, measurements variation, and study setting differences. For example, most of the studies were used different types of measurements tools to assess dietary practice this might be a reason for this finding variation.

Even though the health sectors of Ethiopia developed the health and nutrition policies, strategies and programs, mainly for those of high-risk groups of the population like pregnant women [23, 24], there is a high prevalence of poor dietary practice among pregnant women in this study area. Regarding specific dietary practices like skipping meals, additional meals, avoiding foods, the habit of eating snacks was suboptimal in this study comparing with others previous studies [6, 17]. According to this study, around 21.7% of the study participants had the habit of skipping meals during pregnancy. This study finding is much lower than the study finding from Bahir Dar city [6] and Gedeon zone [17], which indicated around 61.7 and 65.2% of the pregnant women had the habit of skipping meals during pregnancy respectively. On the contrary, in this study around 53.9 and 46.5% of the study participants had no the habit of eating additional meals and snacks between meals during pregnancy respectively, which were much higher than the study finding from Bahir Dar city, which showed that 29.2 and 19.8% of the study participants were not had the habit of eating additional meals and snacks between meals respectively [6]. However, regarding the daily consumption of fresh fruits, vegetables and avoiding the habit of specific food items of these study findings were comparable with the study finding from both Bahir Dar city [6] and Gedeon zone [17].

According to this study finding, the resident of the respondent identified as a strong statistical association (*P* < 0.001) with dietary practice. The respondents from the urban residents were 2.64 times more likely to have good dietary practices than the rural residents of the respondents. This study finding is consistent with the study finding of Guto Gida Woreda of East Wollega zone [28], which revealed that the pregnant women who lived in urban areas were a 49.7 times higher chance of having good nutrition practice than those who lived in a rural area during pregnancy. This might be due to the urban resident could easily access nutrition information and more motivation to take the balanced diet during pregnancy than the rural resident respondent.

In the present study, the average monthly income of the respondent showed a strong statistical association (*P* < 0.001) with dietary practice. The study participants who earn an average monthly income of greater than 2000 ETB were 2.47 times more likely to have good dietary practice than those who earn a monthly income of less than 1000 ETB. This study finding is consistent with the study finding of Ambo [13], Gedeo zone [17], Bahir-Dar city [6], and Addis Ababa [16]. The possible explanation for this could be, the high earned women might easily afford and consume different foods items.

Moreover, this study identified dietary knowledge as a strong statistical association with the dietary practice of the study participants (*P* < 0.001). The respondents who have good dietary knowledge were 2.79 times more likely to have good dietary practice than those who have poor dietary knowledge during pregnancy. This study finding is supported by the study findings of Gondar [15], Gedeo zone [17], Mish [18], and Bahir-Dar city [6] which indicated that the pregnant women who had good dietary knowledge were positively associated with good dietary practice than those who had poor dietary knowledge. This means that as the dietary knowledge of pregnant women increased, the likelihood of good dietary practice of pregnant women could be increased. In addition, in this study nutrition information also showed a statistically significant association (*P* < 0.05) with dietary practice in this current study. The respondents who have nutrition information were 2.5 times higher chance to have good dietary practice than those who have no nutrition information. This finding is consistent with the study finding in Gondar [15], Guto Gida [33], and Ambo [13], which showed that the pregnant women who had nutrition information were positively associated with good dietary practice than those who had no nutrition information during pregnancy.

According to this study, mother occupation showed a statistically significant association (*P* < 0.05) with the dietary practice of the respondents. The government employed mothers were 1.88 times more likely to have good dietary practice than the housewife occupation of women. This study finding is consistent with the study from Misha woreda [18], which showed that the mothers who engaged in government work were seven times more likely to practice good dietary practices than a housewife. This might be due to the reason that those government worker mothers may have more information access about the recommended practice of dietary practice of pregnant women and they may adhere to practice more than the housewives.

In addition, family size also showed a statistical association (*P* < 0.01) with dietary practice. The respondent with a family size of less than 5 was 3.37 times more likely to have good dietary practice than greater than 5 family size. This is similar to the study finding in Guto Gida, which indicated that the pregnant women with a family size of 5 and above had less likely to have good nutrition practice than 1–2 family size [33]. This indicates that as the family members of the households increased, the burden of the pregnant women's dietary habits may be negatively affected.

### Limitation of the study

One of the main limitations of this study was the lack of standardized measurement tools for dietary practice at the national level. In addition, it was not assess food intakes in terms of specific nutrient eaten and the nature of the study is cross-sectional study design which unable to establish cause-effect relation. The use of relatively large sample size and large study area coverage were the strength of the study.

## Conclusion

Generally, in this study, only 23.7% of pregnant women had good dietary practice in Bench-Sheko and Kaffa zone, which indicated dietary practice in the study area, is suboptimal. According to this study findings urban residency, monthly income > 2000EBR, good dietary knowledge, having nutrition information, family size < 5, and government employed mothers occupation were the main identified predictors of the dietary practice of pregnant women. Therefore, to improve this suboptimal dietary practice and alleviate its determinate, providing in-service training for the health professionals and assigning nutritionist to each hospital to provide health and nutrition education as well as should be strengthening the existed nutrition counseling service for pregnant women especially increasing through the outreach community service to address the rural pregnant women. Moreover, the government should also work on sustainable income generation activities to increase the average monthly income level of the study respondents.

## Data Availability

All data are available within the manuscript. Additional data can be obtained from the corresponding author on reasonable request.

## Abbreviations

ANC: Antenatal Care
AOR: Adjusted Odds Ration
COR: Crude Odds Ratio
CI: Confidence Interval
ETB: Ethiopian Birr
HFIAS: Household Food Insecurity Access Scale
SE: Standard Error
SNNPR: South, Nation, Nationality, And People Region
SPSS: Statistical Package Of Social Science
WHO: World Health Organization
